# Predictive value of high-sensitive troponin t versus conventional biomarkers for 1-year left ventricular function and infarct size after STEMI

**DOI:** 10.1186/1532-429X-17-S1-P178

**Published:** 2015-02-03

**Authors:** Sebastian J Reinstadler, Gert Klug, Hans-Josef Feistritzer, Alexander Tu, Michael Schocke, Wolfgang-Michael Franz, Bernhard Metzler

**Affiliations:** University Clinic of Internal Medicine III, Cardiology and Angiology, Medical University Innsbruck, Innsbruck, Austria; Departement of Radiology I, Medical University of Innsbruck, Innsbruck, Austria

## Background

Data relating high-sensitive troponin T (hs-cTnT) to long-term myocardial function and damage in patients after STEMI are lacking. We evaluated the use of serial and peak concentrations of hs-cTnT versus creatine kinase (CK), lactate dehydrogenase (LDH) and high sensitive C-reactive protein (hs-CRP) for prediction of myocardial function as well as infarct scar assessed by cardiac magnetic resonance imaging (CMR) one year after first ST-segment elevation myocardial infarction (STEMI).

## Methods

Sixty-six patients receiving primary percutaneous coronary intervention (p-PCI) for first STEMI were enrolled in this single-centre, observational study. All participants underwent cine and contrast-enhanced CMR within the first week and 12 months after the index event. Serial biomarkers were determined on admission, 6 h, 12 h, 24 h, and 12 months following p-PCI. Hs-cTnT concentrations were measured by a fourth generation high-sensitive immunoassay (Roche Diagnostics^®^). Other biomarkers were assessed using commercially available assays.

## Results

Except for admission values, all single time point and peak hs-cTnT concentrations showed moderate to good correlations with 12-months left ventricular ejection fraction (LVEF) (r = -0.41 to -0.52, all p < 0.01) and infarct size (IS) (r = 0.50 to 0.70, all p < 0.01). Peak CK (LVEF: r = -0.45 to -0.56, IS: r = 0.61 to 0.65), peak LDH (LVEF: r = -0.51 to -0.59, IS: r = 0.54 to 0.69), and peak hs-CRP (LVEF: r = -0.32 to -0.36, IS: r = 0.31 to 0.36) were also significantly related with CMR parameters (all p < 0.05). In receiver-operator characteristic analysis, peak hs-cTnT (AUC = 0.82 (0.71 - 0.92) and 0.89 (0.81 - 0.97), respectively) and peak LDH (AUC = 0.83 (0.74 - 0.93) and 0.91 (0.84 - 0.99), respectively) displayed the best performance for prediction of reduced LVEF (< 55 %, n = 29) and large infarct size at follow-up (> 8 % of left ventricular myocardial mass, n = 36). The combination of biomarkers did not significantly improve the predictive power of hs-cTnT alone (p > 0.05).

## Conclusions

In patients with first STEMI, peak concentrations of hs-cTnT are closely correlated to long-term myocardial function and infarct size. Maximum concentrations of CK, LDH, and hs-CRP were also correlated with 12-months LVEF and infarct size, but did not add any significant prognostic value compared to hs-cTnT alone.

## Funding

Austrian Society of Cardiology.

